# Histone 3 lysine 4 monomethylation supports activation of transcription in *S. cerevisiae* during nutrient stress

**DOI:** 10.1007/s00294-022-01226-2

**Published:** 2022-01-18

**Authors:** Neha Deshpande, Rachel Jordan, Michelle Henderson Pozzi, Mary Bryk

**Affiliations:** 1 Department of Biochemistry and Biophysics, Texas A&M University, 2128 TAMU, College Station, TX 77843 USA; 2 iBio, 8800 HSC Blvd, Bryan, TX 77807 USA

**Keywords:** Histone H3, Chromatin, Methyltransferase, H3K4me1

## Abstract

Mono-methylation of the fourth lysine on the N-terminal tail of histone H3 was found to support the induction of RNA polymerase II transcription in *S. cerevisiae* during nutrient stress. In *S. cerevisiae*, the mono-, di- and tri-methylation of lysine 4 on histone H3 (H3K4) is catalyzed by the protein methyltransferase, Set1. The three distinct methyl marks on H3K4 act in discrete ways to regulate transcription. Nucleosomes enriched with tri-methylated H3K4 are usually associated with active transcription whereas di-methylated H3K4 is associated with gene repression. Mono-methylated H3K4 has been shown to repress gene expression in *S. cerevisiae* and is detected at enhancers and promoters in eukaryotes. *S. cerevisiae set1Δ* mutants unable to methylate H3K4 exhibit growth defects during histidine starvation. The growth defects are rescued by either a wild-type allele of *SET1* or partial-function alleles of *set1*, including a mutant that predominantly generates H3K4me1 and not H3K4me3. Rescue of the growth defect is associated with induction of the *HIS3* gene. Growth defects observed when *set1Δ* cultures were starved for isoleucine and valine were also rescued by wild-type *SET1* or partial-function *set1* alleles. The results show that H3K4me1, in the absence of H3K4me3, supports transcription of the *HIS3* gene and expression of one or more of the genes required for biosynthesis of isoleucine and valine during nutrient stress. Set1-like methyltransferases are evolutionarily conserved, and research has linked their functions to developmental gene regulation and several cancers in higher eukaryotes. Identification of mechanisms of H3K4me1-mediated activation of transcription in budding yeast will provide insight into gene regulation in all eukaryotes.

**Supplementary Information:**

The online version contains supplementary material available at 10.1007/s00294-022-01226-2.

## Introduction

**Fig. 1:**
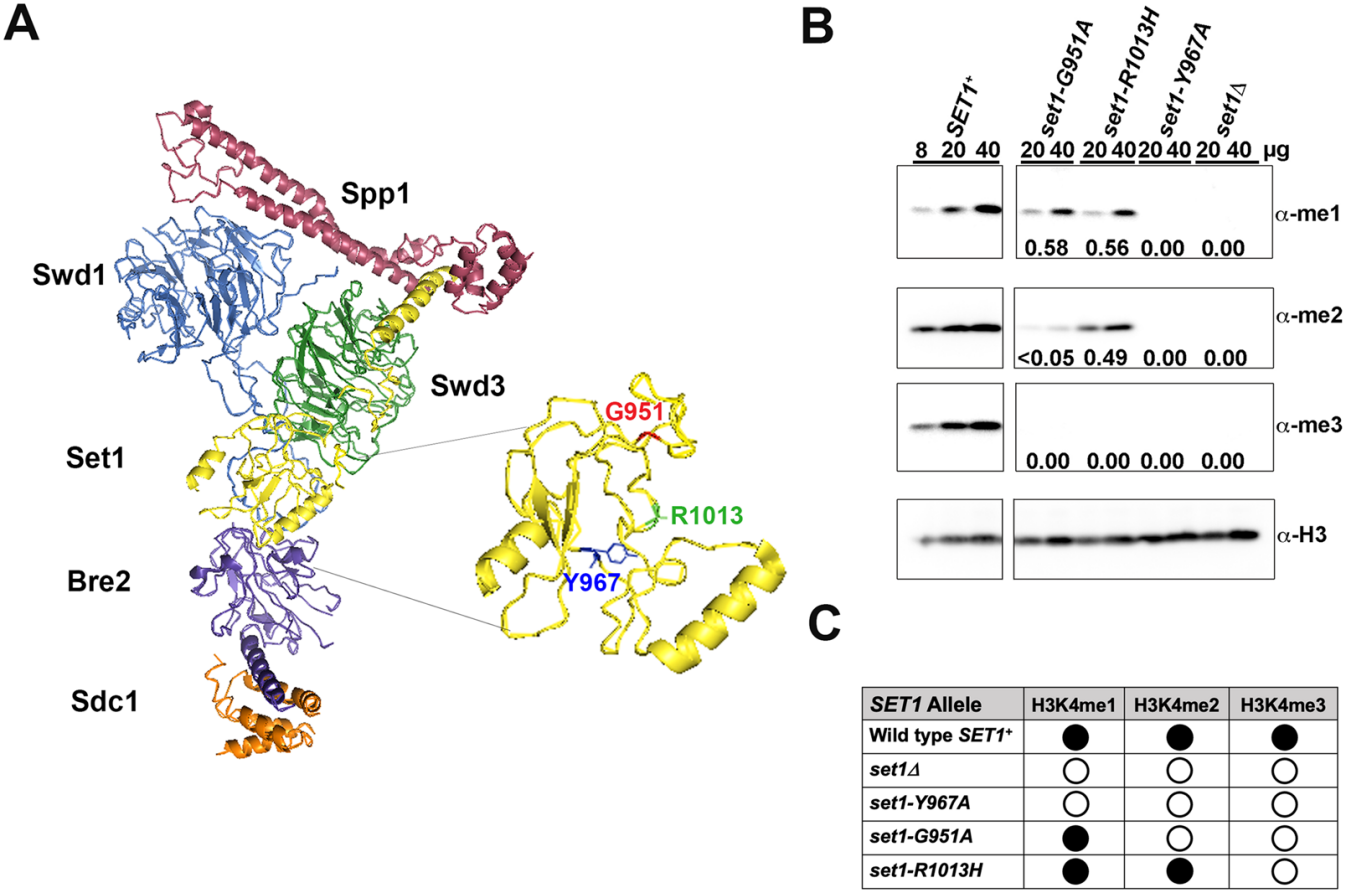
Location and methylation activity of Set1 mutants. **A** Cryo-EM structure of *S. cerevisiae* Set1 in the COMPASS complex (Qu et al. 2018). COMPASS proteins, Bre2 (purple), Sdc1 (orange), Set1 (yellow), Spp1 (magenta), Swd1 (blue), and Swd3 (green) are shown (Krogan et al. 2002; Miller et al. 2001; Nagy et al. 2002; Roguev et al. 2001; Takahashi et al. 2011). The C-terminal tail of Swd1 weaves within the complex to provide a central scaffold for assembly of COMPASS (Qu et al. 2018; Takahashi et al. 2011). The Swd2 protein (not shown) connects Swd1 and Swd3 to the N terminus of Set1 (Wang et al. 2018) and interacts with the CTD of RNA Pol II allowing COMPASS to move along DNA sequences during Pol II transcription (Bae et al. 2020). Inset, substitution of three amino acid (G951, Y967, and R1013) in or near the Set1 active site generated *set1* mutants with different methylation abilities (Williamson et al. 2013). **B** Representative Western blot measuring in vivo steady-state levels of H3K4me1 (α-me1), H3K4me2 (α-me2), and H3K4me3 (α-me3) in whole cell extracts from the yeast strains indicated above the blot image (*n* = 3). The level of total histone H3 (α-H3) was used to normalize the amount of extract loaded in each lane. Numbers indicate the normalized levels of H3K4me1/2/3 detected in the mutants relative to the wild-type *SET1*^+^ strain. Bands labeled 0 (no signal) or > 0.05% (low signal, yet visible by eye) were not detected above background by the Imagequant TL 8.1 image detection software. The samples shown were separated on the same gel and analyzed on the same membranes. Samples between the *SET1*^+^ and *set1-G951A* samples were cropped out of the figure. **C** Summary of the H3K4 methylation activity in wild-type *SET1* and *set1* mutants, a filled circle indicates methylation is detectable above background; open circle, methylation is not detected by Imagequant TL 8.1 image detection software (see “Materials and methods”)

Set1 is the sole H3K4 histone methyltransferase (HMTase) in *S. cerevisiae* that catalyzes the mono-, di- and tri-methylation of the fourth lysine on the amino terminal tail of histone H3 (Briggs et al. 2001; Qu et al. 2018; Shilatifard 2012). Two amino acid substitution mutants of *set1* that encode partial-function H3K4 HMTases, *set1-G951A*, which predominantly mono-methylates H3K4, and *set1-R1013H* that mono- and di-methylates H3K4, were studied to learn about the roles of individual H3K4 methyl marks in transcription by RNA polymerase II in *S. cerevisiae* (Fig. 1). The SET family of H3K4 histone methyltransferases is conserved in eukaryotes (Miller et al. 2001; Takahashi et al. 2011). Mutations in Set1-like H3K4 HMTases alter segmentation in *Drosophila melanogaster* and floral development in *Arabidopsis thaliana* (Breen 1999; Jiang et al. 2011; Shilatifard 2012). Human homologs of Set1, including MLL1 and its translocation alleles, are implicated in hematological malignancies, such as mixed lineage leukemia, acute myeloid leukemia and acute lymphoblastic leukemia (Kandoth et al. 2013; Roguev et al. 2001; Ruault et al. 2002; Shilatifard 2012; Slany 2009). The importance of Set1 family proteins in biological processes from yeast to humans underscores their importance in gene regulation (Cenik and Shilatifard 2021).

The structural organization of eukaryotic DNA into chromatin regulates transcription by RNA polymerases (Han and Grunstein 1988; Izban and Luse 1992; Kornberg and Thonmas 1974; Wasylyk and Chambon 1979; Workman and Kingston 1998). To overcome the physical barrier imposed by chromatin, RNA polymerases rely on *trans*-acting proteins and protein complexes, including chromatin remodelers, transcription factors, co-activators and histone-modifying enzymes to make DNA sequences in chromatin accessible to RNA polymerase II (Castillo et al. 2017; Chatterjee et al. 2011; Côté et al. 1998; Lee et al. 1993, 2007; Santos-Rosa et al. 2003). Methylation of histones by histone methyltransferases regulates transcription by RNA polymerase II (Hyun et al. 2017).

*Saccharomyces cerevisiae* Set1 is a member of the Complex Associated with Set1, COMPASS (Fig. 1) (Bae et al. 2020; Briggs et al. 2001; Miller et al. 2001; Morillon et al. 2005; Mueller et al. 2006; Roguev et al. 2001). The effect of H3K4 methylation on the accessibility of chromatin depends on the chromatin context, consistent with published work showing that H3K4 mono-, di-, and tri-methylation have different effects on gene transcription (Kusch 2012; Pokholok et al. 2005). Chromatin immunoprecipitation (ChIP) and ChIP-seq experiments revealed that the distribution of H3K4me1, H3K4me2 and H3K4me3 across open reading frames is not identical (Bernstein et al. 2005; Pokholok et al. 2005; Soares et al. 2017; Weiner et al. 2012). For the most highly expressed genes in *S. cerevisiae*, nucleosomes with H3K4me3 peak at the promoter and up to ~ 200 bp beyond the transcription start site, nucleosomes with H3K4me2 are enriched in the middle of the ORF, and nucleosomes with H3K4me1 are found at relatively low levels across an ORF. In some cases, the distributions of H3K4 methylated histones have provided insight into their functions.

Methylation of histones on its own has not been shown to change the structure of chromatin, instead methylated histones may act by recruiting effector proteins to chromatin (Cheng et al. 2014; Musselman et al. 2012; Pray-Grant et al. 2005; Taverna et al. 2006). H3K4me3 is usually associated with active transcription (Kusch 2012; Ng et al. 2003; Schneider et al. 2005), which is supported by work showing that H3K4me3 is recognized by the chromodomain-containing protein, Chd1, a member of the SAGA transcription coactivator complex (Pray-Grant et al. 2005). The transcription factor TAF3 also interacts with H3K4me3 to recruit TF_II_D to promoters (Vermeulen et al. 2007). In *S. cerevisiae*, the chromatin remodeler Isw1 interacts with methylated H3K4 to generate accessible chromatin at the 5′ end of the *MET16* gene (Santos-Rosa et al. 2003). H3K4me3 is also recognized by other protein complexes, some of which are negative effectors of transcription (Musselman et al. 2012). In response to DNA damage, the PHD domain of ING2 (INhibitor of Growth 2), a subunit of the mSin3a–HDAC1 histone deacetylase complex, binds to H3K4me3, stabilizing mSin3a–HDAC1 at the promoter of cyclin D1 gene and other proliferation genes, leading to repression of transcription (Shi et al. 2006). H3K4me2 is associated with repression of transcription and has been shown to interact with histone deacetylases that deacetylate histones at the 5′ ends of some highly expressed genes (Kim and Buratowski 2009; Pinskaya and Morillon 2009).

Progress is being made toward understanding the roles H3K4me1 plays in gene regulation. In yeast, the absence or presence of H3K4me1 at osmostress-responsive gene determines whether RSC or SWR-C remodels chromatin at the promoters (Nadal-Ribelles et al. 2015). In higher eukaryotes, nucleosomes with H3K4me1 are enriched at enhancer elements and the function of H3K4me1 at enhancers is a topic of intense research (Calo and Wysocka 2013; Catarino and Stark 2018; Froimchuk et al. 2017). Recent work using human and mouse germ cells and ESCs showed that the patterns of H3K4me1, H3K4me3 and H3K27me3 at promoters specify the potential transcriptional state of a promoter (Bae and Lesch 2020). The ability to tease apart the roles of individual H3K4 methyl marks will lead to better understanding of the role of H3K4me1 in gene expression.

Because Set1 is the only H3K4 HMTase in *S. cerevisiae*, it is an excellent system to study the effect of the three H3K4 methyl marks on transcription. In a previous study, mutants of *SET1* were made that encode proteins with amino acid substitutions in the SET domain of Set1 (Fig. 1) (Williamson et al. 2013). Amino acid substitution mutants were constructed to alter residues near and in the active site of Set1 to generate mutants with different H3K4 methylation capabilities. Yeast strains expressing the *set1-Y967A* allele are indistinguishable from a *set1Δ* mutant with respect to methylation of H3K4. The *set1-G951A* mutant produces predominantly H3K4me1 with low levels of H3K4me2. The residue G951 is highly conserved in Set1 family proteins and is important for Set1 function (Dillon et al. 2005; Lee et al. 2018; Nislow et al. 1997; Sollier et al. 2004). A second partial-function mutant, *set1-R1013H*, generates H3K4me1 and H3K4me2 in vivo (Fig. 1).

In this study, *set1* partial-function mutants were studied to learn about the roles of individual H3K4 methyl marks in RNA polymerase II transcription in *S. cerevisiae*. Two amino acid biosynthetic pathways, the histidine and isoleucine–valine pathways, were used to study the roles of individual H3K4 methyl marks in transcription when cells are starved for amino acids. The results show that induction of the *HIS3* gene occurs in the absence of H3K4me2 and H3K4me3, indicating that H3K4me1 supports induction of the *HIS3* gene under histidine-starvation conditions. In addition, the results suggest that one or more genes required for biosynthesis of isoleucine and valine (Falco and Dumas 1985; Falco et al. 1985) is activated in the presence of H3K4me1 when H3K4me3 and H3K4me2 are absent. The major finding is that H3K4me1 supports induction of transcription in *S. cerevisiae* cultures grown under amino-acid starvation conditions.

### Materials and methods

#### Media

Standard media preparation protocols were used (Rose et al. 1990). YPADTU is YPD media supplemented with 40 mg/L adenine hemisulfate, 80 mg/L L-tryptophan and 20 mg/L uracil. Where indicated, 1 M 3-amino-1,2,4-triazole (3AT) made in sterile H_2_O was added to media to a final concentration of 10 mM. Sulfometuron Methyl (SMM), made in dimethyl sulfoxide (DMSO), was added to media to a final concentration of 1 μg/mL. SC Complete is defined synthetic medium containing all nutrients required for yeast cell growth. SC-His, SC-Ile Val and SC-Trp are synthetic complete yeast growth media lacking histidine, isoleucine and valine, or tryptophan, respectively.

### Yeast strains

*Saccharomyces cerevisiae* strains used in this study are listed in Supplementary Table 1. Yeast strains were made by standard genetic crosses and genetic transformation. The initial characterization of mutant alleles of *SET1* was described previously (Williamson et al. 2013). Cloned *SET1*^+^ or *set1* mutant genes in *Stu*I-linearized pRS406 plasmids were integrated into the *ura3-52* locus of MBY1590 and MBY3078 to make the *SET1*^+^ (MBY2994 and MBY3148, respectively), *set1-Y967A* (MBY2998 and MBY3152, respectively), and *set1-R1013H* (MBY2996 and MBY3181, respectively) strains (Sikorski and Hieter 1989). The cloned *set1-G951A* gene in a *Stu*I-linearized pRS406 plasmid was integrated into the *ura3-52* locus of MBY3078 to make MBY3154. A *Stu*I-linearized pRS406 plasmid with no insert was integrated into the *ura3-52* locus of MBY1590 to make MBY2992 and into the *ura3-52* locus of MBY3078 to make MBY3149.

Plasmids containing *HHT2*-*HHF2* and *hht2*-K4R-*HHF2* have been described previously (Briggs et al. 2001). The genes encoding *HHT2* and *HHF2* or *hht2*-K4R-*HHF2* were cloned into pRS414 plasmids that carry a *TRP1* selectable marker (Sikorski and Hieter 1989). The endogenous *S. cerevisiae HHT1-HHF1* and *HHT2-HHF2* genes encoding histones H3 and H4 were deleted and replaced with selectable marker genes. The resulting yeast strains expressed either the wild-type *HHT2-HHF2* genes (p*HHT2-HHF2 TRP1 CEN*, MBY3029) or a mutant version of *HHT2 (hht2-H3K4R)* with a wild-type *HHF2* gene (p*hht2-K4R-HHF2 TRP1 CEN*, MBY3030). Cloned *SET1*^+^ or *set1* mutant genes in *Stu*I-linearized pRS406 plasmids were integrated into the *ura3-52* locus of MBY3029 to make the *SET1*^+^ (MBY3031), *set1-G951A* (MBY3242), *set1-Y967A* (MBY3033), or *set1-R1013H* (MBY3035) strains. A *Stu*I-linearized pRS406 plasmid with no insert was integrated into the *ura3-52* locus of MBY3029 to make the *set1Δ* strain (MBY3032). The *Stu*I-linearized plasmids were also transformed into MBY3030 (with p*hht2-K4R-HHF2* genes) to make the *SET1*^+^ (MBY3037), *set1-G951A* (MBY3243), *set1-Y967A* (MBY3039), *set1-R1013H* (MBY3041) and the *set1Δ* (MBY3038) strains.

### Molecular rendering of COMPASS

The structure of the COMPASS complex from PDB: 6BX3 (Qu et al. 2018) was modified using PyMOL (v.1.7.4.5 Schrodinger (2015)) to highlight modified amino acid residues in the Set1 protein (Fig. 1).

### Growth assays

Cultures were grown to saturation at 30 °C in SC-His or YPADTU liquid medium. Eight, five-fold serial dilutions were made in sterile water. The last six dilutions were plated (5 µl) on each of four types of solid agar plates: SC-His, SC-His + 10 mM 3AT, SC-complete, and SC-complete + 10 mM 3AT. Plates were imaged after 24 and 42–44 h of incubation at 30 °C. For experiments with yeast strains expressing histone H3K4 or H3K4R from plasmids, six ten-fold serial dilutions were made in sterile water and dilutions were plated, as described above. Plates were incubated at 30 °C and imaged after 42 h. For Ile Val starvation growth assays, eight, five-fold serial dilutions were made in sterile water using overnight cultures grown in YPADTU. The last six dilutions were plated (5 µl) on solid agar plates: SC-Ile Val + DMSO, SC-Ile Val + 1 μg/mL SMM, SC-complete + DMSO, and SC-complete + 1 μg/mL SMM. Plates were imaged after 72–96 h of incubation at 30 °C.

### RNA isolation and northern blotting

Saturated cultures of yeast strains grown in SC-His liquid medium were diluted into 50 mL of fresh SC-His medium at ~ 4 × 10^6^ cells/mL and grown to a density of 1–2 × 10^7^ cells/mL at 30^o^ C in a shaker incubator. After 4 h, 500 μL of 1 M 3AT (final concentration 10 mM) or sterile distilled water were added to the cultures, which were incubated at 30^o^ C in a shaker incubator for one hour. Total RNA was extracted from yeast cultures, as previously described (Schmitt et al. 1990) with the following modifications. Cells were re-suspended in 450 µl AE buffer (50 mM C_2_H_3_NaO_2_ pH 5.3, 10 mM EDTA), and then transferred to a microfuge tube to which 50 µl 10% SDS was added. Extraction with an equal volume of chloroform:isoamyl alcohol (24:1) was performed before the addition of 50 µL 3 M sodium acetate pH 5.3 and 2.5 volumes 100% ethanol. After precipitation, RNA was re-suspended in sterile milliQ H_2_O and stored at − 70 °C. RNA (15 µg) was analyzed by Northern blotting, as described previously (Swanson et al. 1991). The steady-state level of *HIS3* transcript was detected by hybridization with a strand-specific ^32^P-labeled riboprobe. To normalize loading of RNA samples, a ^32^P-labeled *ACT1* DNA probe synthesized by random priming was used to detect the *ACT1* mRNA level. For the northern blots analyzing the *set1-G951A* strain, a ^32^P-labeled *HIS3* DNA probe synthesized by random priming was used to detect the *HIS3* mRNA level. To analyze the Northern blots, the ratio of *HIS3*/*ACT1* mRNA was calculated for each strain grown in SC-His and SC-His + 10 mM 3AT. To calculate the fold change of *HIS3* transcript in presence of 3AT, the ratio of *HIS3*/*ACT1* mRNA for each strain grown in SC-His + 10 mM 3AT was normalized to the corresponding *HIS3*/*ACT1* transcript levels from cultures grown in SC-His.

The optimal induction of *HIS3* transcript after addition of 3AT was determined using a time-course experiment (Supplementary Fig. 1). The steady-state level of *HIS3* transcript was detected as described above. To normalize loading of RNA samples, a ^32^P-labeled 18S ribosomal RNA riboprobe was used to detect the rRNA level. To analyze the Northern blots, the ratio of *HIS3* mRNA/18S rRNA was calculated for each strain at each time point. Then, the ratio of *HIS3* mRNA/18S rRNA at 5, 10, 30, 60, and 120 min post addition of 10 mM 3AT was normalized to the corresponding ratio at 0 min (prior to adding 3AT). All Northern blots were imaged using a G.E Typhoon FLA 7000 and quantified using G.E Imagequant TL 8.1 software.

### Whole cell protein extracts and western blotting

Yeast whole cell protein extracts were prepared as described (Mueller et al. 2006). Proteins from clarified whole-cell extracts (8, 20 or 40 μg) were separated on 10% SDS–polyacrylamide gels, transferred to PVDF membrane, and probed with α-histone H3 (ab1791, Abcam; 1:1000), α-K4-monomethyl H3 (13-0040, Epicypher, 1:2000), α-K4-dimethyl H3 (710796, Invitrogen; 1:1000), or α-K4-trimethyl H3 (13-0041, Epicypher; 1:2000). Antibody binding was detected with HRP-conjugated α-rabbit secondary antibodies (1706715, Biorad; 1:2000) and Clarity Western ECL substrate (Bio-Rad, Hercules, CA, USA). Western blots were imaged on an Amersham Imager 600 and quantified using Imagequant TL 8.1 software.

### Chromatin immunoprecipitation

Saturated cultures of yeast strains grown in SC-His liquid medium were diluted into 200 mL of fresh SC-His medium at ~ 4 × 10^6^ cells/mL and grown to a density of 1–2 × 10^7^ cells/mL at 30^o^ C in a shaker incubator. After 4 h, 2 mL of 1 M 3AT (final concentration 10 mM) or sterile distilled water was added to the cultures, which were incubated at 30^o^ C in a shaker incubator for 60 min. Lysates were prepared as previously described (Li et al. 2006) with the following modifications. Breakage of cells was performed in 500 μL lysis buffer (50 mM HEPES/KOH pH 7.5, 140 mM NaCl, 1 mM EDTA, 1% Triton X-100, 0.1% Sodium deoxycholate, 1 mM PMSF, 1 mM benzamidine, 1 μg/mL each leupeptin pepstatin, and bestatin) using a Mini-BeadBeater 16 (Biospec) at 4 °C with 1 min beating followed by 2 min of rest, repeated four times. Chromatin in 1 mL of lysis buffer was sonicated in a 4 °C water bath (Bioruptor Water Cooler, Diagenode) using a Bioruptor 300 Sonication System (Diagenode) for 300 cycles of 30 s on, 45 s off, power setting high, to shear chromatin to a length ≤ 500 bp. Sonicated chromatin was clarified by centrifugation at 13 K rpm, 4 °C, 30 min. Sonicated chromatin was incubated with antibody in a total volume of 500 μL for 16–18 h with rocking at 4 °C. The following antibodies were used; α-H3, Abcam ab1791 (4 µg/IP, lot #s: GR3297884-1, GR3297878-1, GR3356864-1 and GR3366670-1), α-H3K4me3, Epicypher 13-0041 (2 µg/IP, lot #s: 20083002-42 and 20218003-49), α-H3K4me2, Epicypher 13-0027 (4 µg/IP lot #: 20252002-04), α-H3K4me1, Epicypher 13-0040 (2 µg/IP lot #s: 19338001-42 and 20178005-44). IPs were processed as described previously (Bryk et al. 2002), with the following exceptions. Pierce protein A/G agarose beads were used to pull down crosslinked protein-DNA complexes (Pierce Biotechnology, Thermofisher, IL, USA). ChIP eluates were purified using the ChIP DNA clean and concentrator kit, as recommended by the manufacturer (Zymo Research Corp, CA, USA). Purified extracts eluted in 100 µL elution buffer were stored at − 70 °C.

### Analysis of ChIPs

Quantitative polymerase chain reactions (qPCR) were performed to analyze the distribution of H3K4me1, H3K4me2, H3K4me3 and total histone H3 at *HIS3* promoter, 5′ and 3′ regions of the *HIS3* ORF, the promoter of the *ACT1* gene, and a 284-bp intergenic region (IGR) on chromosome *VIII* from positions 384,624 to 384,908. Oligonucleotides are listed in Supplementary Table 2.

To analyze the H3K4me marks in ChIP eluates, duplicate reactions using 2.5 μL input DNA (1:10) and 2.5 μL immuno-precipitated DNA were amplified in 10 μL reactions containing 0.5 μM each oligonucleotide and 1X homemade master mix (1X New England Biolabs (NEB) PCR buffer, 2.5 mM MgCl_2_, 0.2 mM dNTPs, 1X Evagreen (Biotium), and 1 unit NEB Taq). Reactions were performed in a BioRad CFX96 Real-Time System C1000 Thermal 71 Cycler. The PCR parameters were 1 cycle of 95 °C, 3 min; 30 cycles of 95 °C for 30 s, 58 °C for 30 s, 72 °C for 30 s; 1 cycle of 95 °C for 30 s followed by melt curve analysis from 65 °C to 95 °C in 0.5 °C increments for 5 s each. For analysis of *HIS3* 3′ ORF, the annealing temperature was 54 °C. The threshold cycle reading was taken after each 72 °C elongation step. Percentage of DNA immuno-precipitated (%IP) was calculated by dividing the signal from IP DNA by that of input DNA for all H3K4me ChIPs and total histone H3 ChIPs from each location. The %IP for each H3K4me mark was normalized to the %IP of total H3 at each location.

### Statistical analysis

Statistical analysis for the Northern blots and ChIP experiments was performed using the Mann–Whitney *U* test (Allaire 2012) on R studio.

## Results

### Histone H3K4 methylation by Set1 is required for robust growth of yeast cultures when grown under nutrient stress

The positions of the amino-acid substitutions in the *set1* mutants used in this study are shown in a reproduction of the COMPASS cryo-EM structure (Qu et al. 2018) (Fig. 1A). The *set1* mutants generate levels of H3K4me1/2/3 that are different from those observed in cultures expressing wild-type *SET1*. Western blots using whole cell extracts show that two mutants, *set1Δ* and *set1-Y967A*, have no detectable H3K4me1/2/3; *set1-G951A* has H3K4me1 at 58% of *SET1*^+^ (WT), H3K4me2 at < 5% of WT and no detectable H3K4me3; and *set1-R1013H* has H3K4me1 at 56% of WT, H3K4me2 at 49% of WT, and no detectable H3K4me3 (Fig. 1B). The methylation proficiency of wild-type *SET1* and the *set1* mutants is indicated in Fig. 1C.

Two amino acid biosynthetic pathways, the histidine and isoleucine–valine pathways, were used as models to evaluate the role of individual H3K4 methyl marks during amino-acid starvation. The *HIS3* gene codes for imidazole glycerol phosphate dehydratase (Fink 1964), the enzyme that catalyzes the sixth step in the biosynthesis of histidine in *S. cerevisiae*. The herbicide 3-amino-1,2,4-triazole (3AT) is a competitive inhibitor of imidazole glycerol phosphate dehydratase and it is used to induce histidine-starvation. Amino acid starvation initiates the general amino acid control (GAAC) pathway in *S. cerevisiae* (Brennan and Struhl 1980; Hope and Struhl 1985). The *HIS3* gene and most amino acid biosynthetic genes are regulated by the GAAC pathway and require the transcription regulator Gcn4 for initiation of transcription (Hinnebusch 2005). Growth of yeast cultures in 3AT activates Gcn4, which recruits the histone acetyltransferase (HAT) Gcn5 to many promoters, including the *HIS3* promoter, initiating a cascade of events that lead to transcription of the *HIS3* gene (Hill et al. 1986; Kuo and Allis 1998; Kuo et al. 2000). The GAAC pathway also controls genes required for the biosynthesis of isoleucine and valine in *S. cerevisiae*. Sulfometuron methyl (SMM) is a competitive inhibitor of the *ILV2* gene, acetolactate synthase (Falco and Dumas 1985). In this study, SMM was used to induce starvation for isoleucine and valine to evaluate the effect of H3K4 methylation on growth of *S. cerevisiae* cultures during starvation for branched-chain amino acids.

**Fig. 2:**
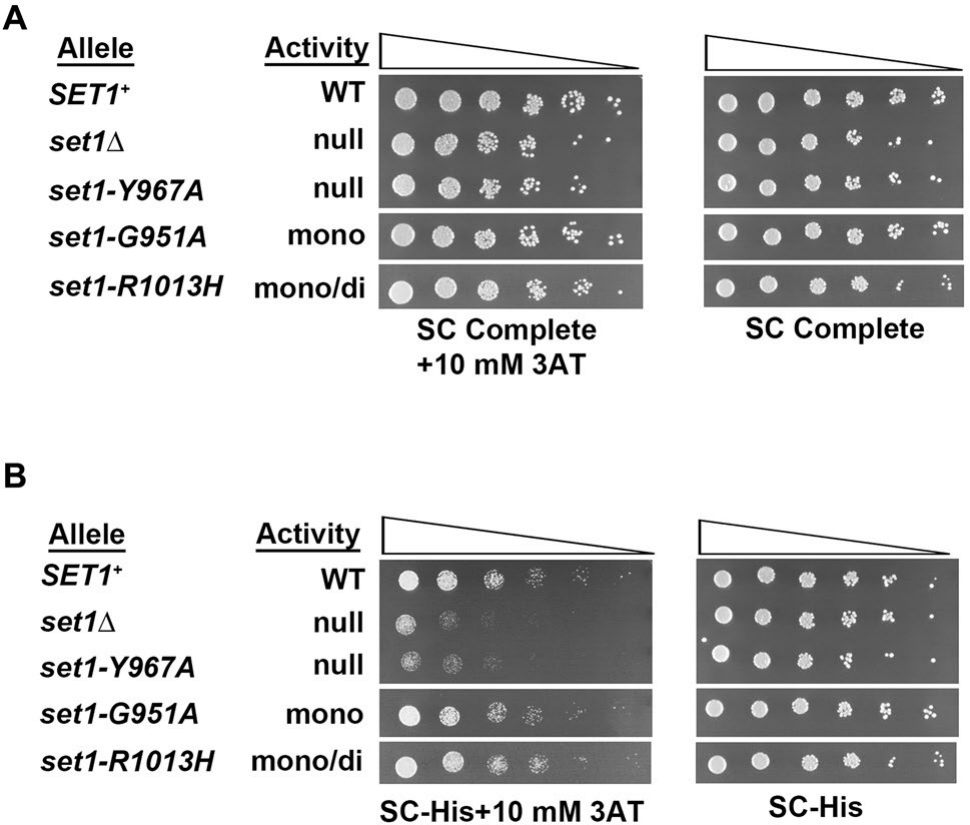
Growth of mutants lacking Set1 methylation activity is sensitive to histidine starvation induced by 3AT. Five-fold serial dilutions of yeast cultures expressing wild-type *SET1*^+^ or *set1* mutant alleles were spotted onto **A** SC-complete agar with or without 10 mM 3AT and **B** SC-His agar with or without 10 mM 3AT. Allele, relevant genotype. Activity, H3K4 methylation activity determined by Western blots (Fig. 1B); WT, H3K4me1/2/3 detected; Null, no H3K4me1/2/3 detected; mono, H3K4me1 detected with low or no H3K4me2/3; mono/di, H3K4me1/2 detected with no H3K4me3 (Fig. 1B). Dilution of the cultures, right triangle at the top of each column. Plates were incubated at 30 °C for 42 h prior to imaging. All cultures shown in the figure were grown on the same plate. The images were cut to remove a strain not being considered here

The effects of alterations in the levels of H3K4me1/2/3 on the growth of yeast cultures under histidine-starvation conditions were evaluated using yeast cultures expressing either wild-type *SET1*, *set1Δ* or one of three *set1* amino-acid substitution variants on four different types of solid agar media (Fig. 2). The cultures grew similarly on SC Complete medium with the herbicide 3AT, SC Complete medium without 3AT, and SC medium lacking histidine, SC-His (Fig. 2A, B right panel). In contrast, all cultures grew less well on SC-His + 3AT (Fig. 2B, left panel). The herbicide 3AT was expected to have a negative effect on growth because it causes histidine starvation when used in medium lacking histidine.

Reduced growth of the *set1Δ* and *set1-Y967A* mutants was observed on SC-His + 3AT agar (Fig. 2B, left panel). The extent of growth of *set1-G951A*, *set1-R1013H, or SET1*^+^ yeast strains expressing partially or fully functional Set1 proteins on SC-His + 3AT agar was similar, and all grew better than the *set1Δ* and *set1-Y967A* mutants. Given that the *set1-G951A* mutant catalyzes H3K4me1 mainly with very low levels of H3K4me2 and undetectable H3K4me3 (Fig. 1B), the results suggest that H3K4me2 and H3K4me3 are not required for wild-type levels of growth under histidine-starvation conditions.

**Fig. 3:**
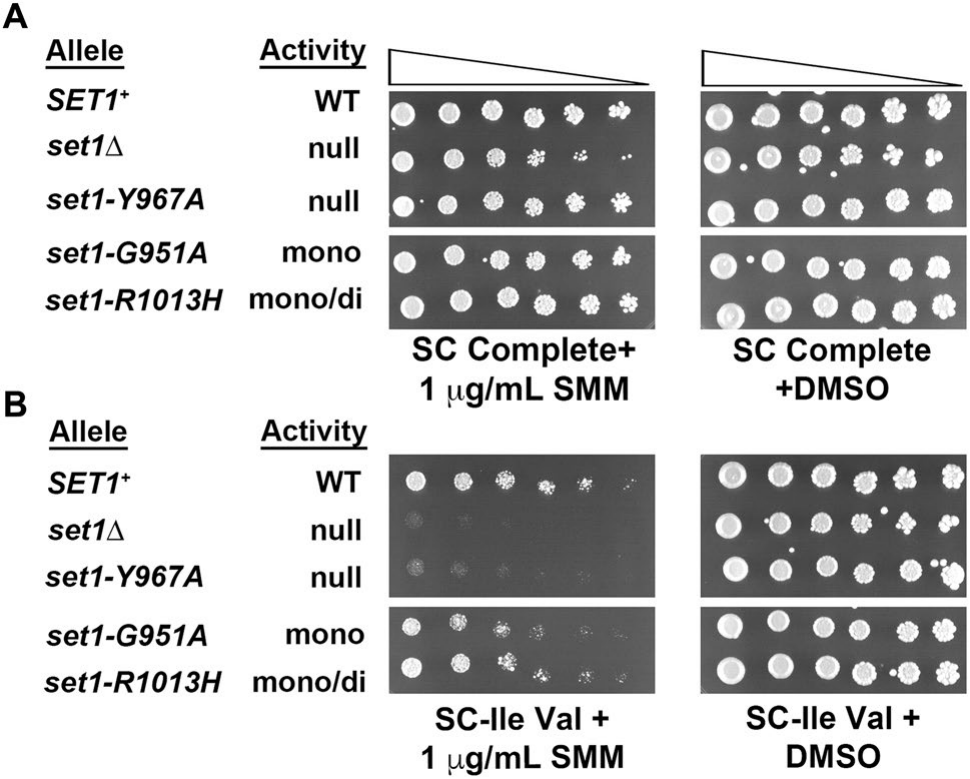
Growth of mutants lacking Set1 methylation activity is sensitive to isoleucine and valine starvation induced by SMM. Five-fold serial dilutions of yeast strains expressing wild-type *SET1* or mutant *set1* alleles were spotted onto **A** SC-complete solid agar and **B** SC-Ile Val plates with 1 µg/mL SMM or DMSO. Plates were incubated at 30 °C for 4–5 days prior to imaging. Other labels, as in Fig. 2. All cultures shown were grown on the same plate. The images were cut to remove a strain not being considered here

A literature search was performed to identify other genes regulated by the GAAC pathway that might be also regulated by H3K4 methylation. The *ILV* genes, *ILV1*, *ILV2*, *ILV3*, *ILV5* and *ILV6* and the aminotransferases *BAT1* and *BAT2*, encode enzymes that catalyze the biosynthesis of isoleucine and valine in *S. cerevisiae*. SMM, a competitive inhibitor of the *ILV2* gene, was used to evaluate the role of H3K4 methylation on growth of cultures during starvation for isoleucine and valine. Saturated cultures of yeast strains expressing wild-type *SET1*, *set1Δ* or *set1* amino-acid substitution variants were analyzed for growth on medium with or without SMM. The growth of strains on SC Complete media + DMSO and SC Complete containing SMM was mostly similar, although the size of *set1Δ* colonies on SC Complete + SMM was smaller than other strains (Fig. 3A). Growth of the five yeast strains was similar on synthetic medium lacking isoleucine and valine (SC-Ile Val + DMSO, Fig. 3B, right). When compared to strains expressing wild-type *SET1*^+^ or the partial-function alleles of *set1* (*set1-G951A* and *set1-R1013H*), the *set1Δ* and *set1-Y967A* mutants grew poorly on medium lacking isoleucine and valine in presence of SMM (SC-Ile Val + 1 μg/mL SMM) (Fig. 3B, left). The partial-function mutant *set1-G951A,* which performs mainly H3K4me1, exhibited better growth than the *set1Δ* and *set1-Y967A* mutants suggesting that H3K4me2 and H3K4me3 are not required for wild-type growth under conditions of isoleucine and valine starvation.

**Fig. 4:**
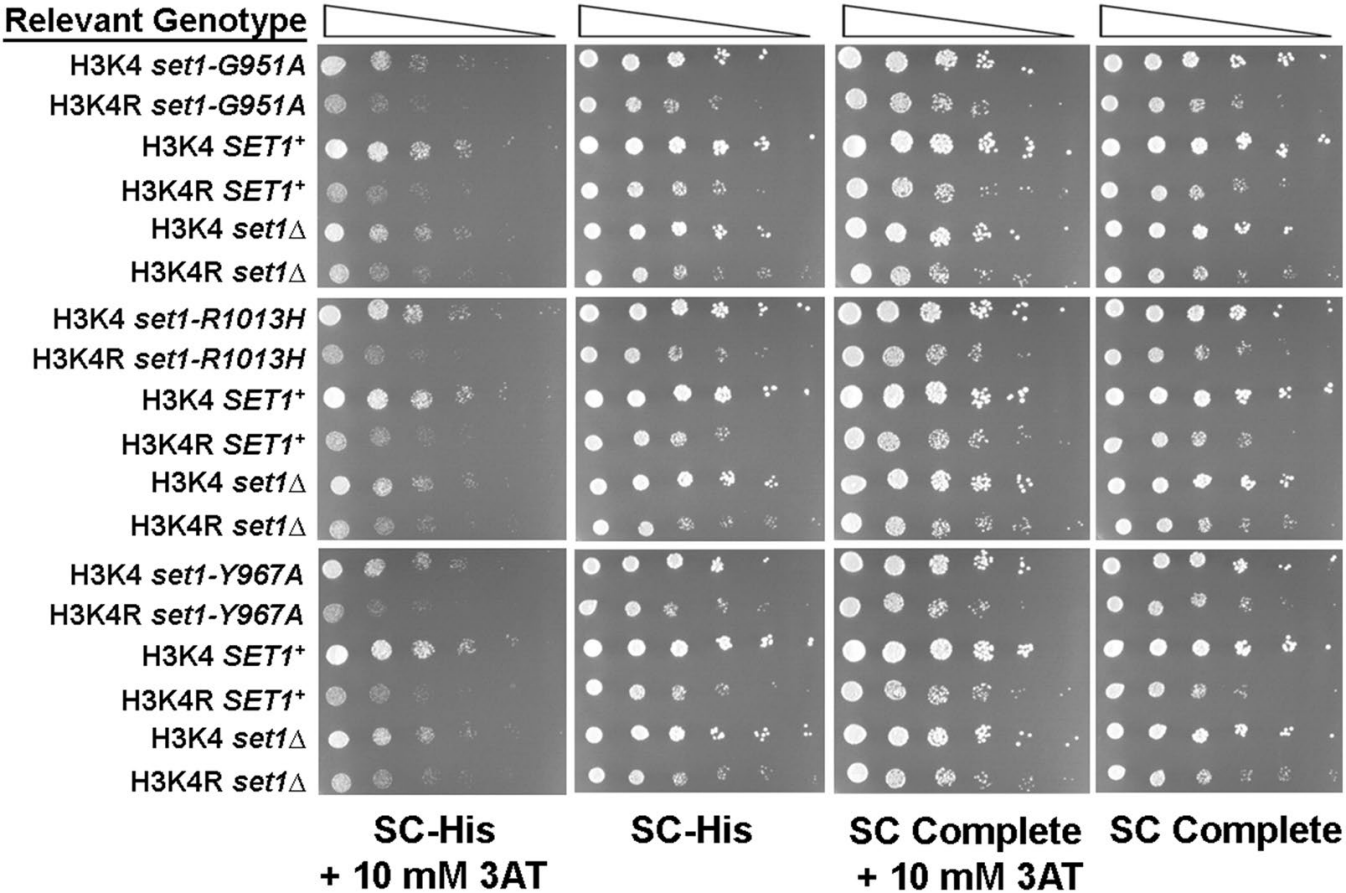
Methylation of histone H3K4 by Set1 is required for robust growth on during histidine starvation. Five-fold serial dilutions of yeast cultures were spotted on to solid media, SC Complete and SC-His, with or without 10 mM 3AT. The relevant genotypes listed on the left indicate yeast strains expressing wild-type or mutant alleles of *SET1* and either wild-type histones H3 and H4 (H3K4) or mutant H3 K4R and wild-type H4, (H3K4R). Plates were incubated at 30 °C for 72 h prior to imaging (*n* = 3). Other labels, as in Fig. 2

In addition to methylation of histone H3, Set1 methylates the Dam1 protein that functions in chromosome segregation in *S. cerevisiae* (Latham et al. 2011; Zhang et al. 2005)*.* To verify that the growth phenotypes observed in Figs. 2 and 3 were due to changes in methylation of histone H3 and not another target of Set1, *SET1*^+^ and *set1* mutant alleles were transformed into yeast strains that express either wild-type histone H3 or a mutant version of H3 (*hht2-K4R*, abbreviated H3K4R) that cannot be methylated by Set1 due to the replacement of lysine at position 4 with arginine (Fig. 4 and Supplementary Fig. 2). These yeast strains express H3 and H4 from a single gene cassette on a centromere-based plasmid. Expression of the un-methylatable K4R variant of histone H3 (H3K4R) caused reduced growth, which can be seen by comparing growth of strains expressing H3K4 to those expressing H3K4R (for example, compare extent of growth of H3K4 *SET1*^+^ and H3K4R *SET1*^+^ on each type of media, Fig. 4). It is clear that methylation of histone H3 by Set1 is required for robust growth during amino acid starvation because the growth of yeast strain expressing un-methylatable H3K4R and *SET1*^+^ (H3K4R *SET1*^+^) was the same as the H3K4R *set1Δ* yeast strain on SC-His + 3AT agar (for example, top left panel, Fig. 4). The results indicate that histone H3 is the relevant target of Set1. Therefore, methylation of H3K4 by Set1 is required for robust growth of yeast cultures during histidine starvation and isoleucine and valine starvation (Supplementary Fig. 2).

As was observed with yeast strains carrying a normal complement of histone H3 and H4 genes (Fig. 2), the growth defect in the *set1Δ* cultures is detected when compared to H3K4 *SET1*^+^ or H3K4 *set1-R1013H* on SC-His + 3AT (Fig. 4). However, the extent of growth of the H3K4 *set1-G951A* culture and the H3K4 *set1Δ* cultures was similar on SC-His + 3AT (Fig. 4, top left panel), indicating the growth defect caused by histidine starvation was lost in *set1-G951A* mutant when coupled with reduced dosage of the histones H3 and H4 genes. One possibility is that in this genetic background with expression of histones H3 and H4 from a single H3-H4 cassette, the loss of H3K4me2 and H3K4me3 causes stronger growth defects under stress conditions. Altered phenotypes caused by expression of the histones H3 and H4 from a single H3-H4 cassette have been observed previously (Clark-Adams et al. 1988; Wyrick et al. 1999; Yu et al. 2019). The role of H3K4 methylation in expression of *HIS3* was explored further by measuring steady-state levels of *HIS3* mRNA and the association of H3K4 methyl marks at the *HIS3* gene in wild-type *SET1*^+^ strains and the hypomorphic *set1* mutants.

### Histone H3K4 methylation by Set1 is required for wild-type expression of the *HIS3* gene in cultures grown in nutrient-stress conditions

**Fig. 5:**
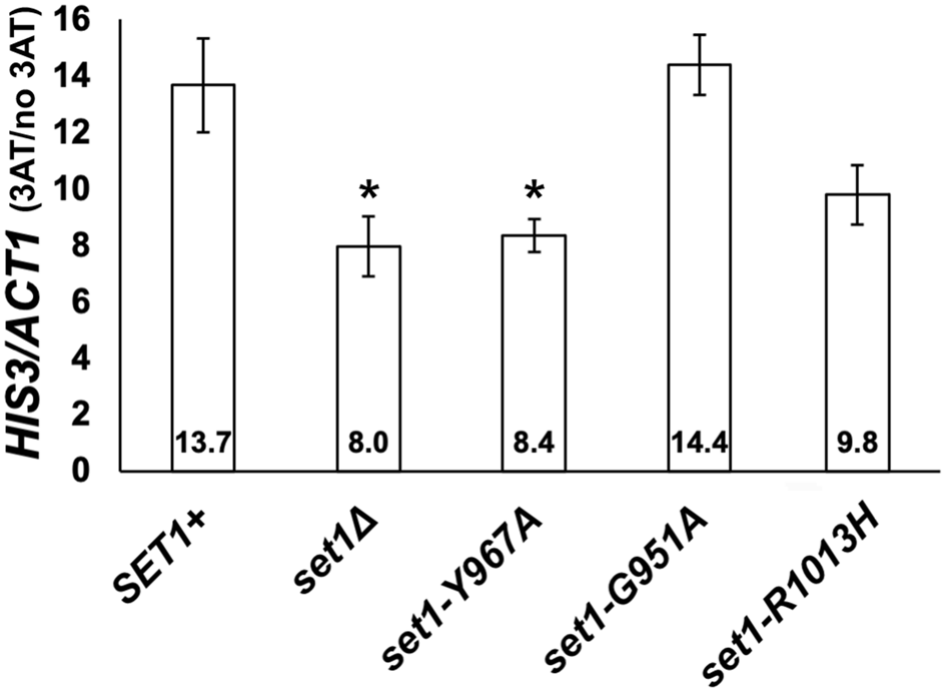
*HIS3* transcript levels are low in *set1* null mutants under histidine-starvation conditions. Bar graph shows the steady-state *HIS3* mRNA levels in total RNA isolated from wild-type strains and *set1* mutants. The *HIS3*/*ACT1* mRNA ratios for cultures grown in SC-His + 10 mM 3AT were normalized to the ratios from the same cultures grown in SC-His (3AT/no 3AT). The values of the 3AT/no 3AT ratios were compared using the Mann Whitney *U* test (Allaire 2012); *SET1*^+^, 13.7 ± 1.7; *set1Δ*, 8.0 ± 1.1; *set1-Y967A*, 8.4 ± 0.6; *set1-G951A*, 14.4 ± 1.1; *set1-Y967F*, 13.5 ± 1.2; *set1-R1013H*, 9.8 ± 1.1. The *set1Δ* and *set1-Y967A* mutants had significantly lower *HIS3* mRNA levels than the wild-type *SET1*^+^ (*p = 0.02 *SET1*^+^ vs. *set1Δ* and *SET1*^+^ vs. *set1-Y967A*). The level of *HIS3* mRNA in the *set1-G951A* mutant was similar to wild-type Set1 and significantly different from the H3K4 methylation null mutants (*p = 0.02 *set1-G951A* vs. *set1Δ*, *p = 0.035 *set1-G951A* vs. *set1-Y967A*). Error bars represent standard error of the mean, SEM (*n* = 6 for *SET1*^+^, *set1Δ* and *set1-Y967F*, *n* = 5 for *set1-Y967A* and *set1-R013H,* *n* = 3 for *set1-G951A*)

The level of *HIS3* mRNA in cultures expressing wild-type *SET1*^+^ or the *set1* amino-acid substitution variants was measured by Northern hybridization (Fig. 5). Histidine starvation was induced by adding 3AT to log-phase cultures for 1 h prior to isolation of RNA. *HIS3* mRNA was increased 13.7-fold in *SET1*^+^ cultures when histidine starvation was induced. In contrast, the increase in the *HIS3* mRNA in the *set1Δ* and *set1-Y967A* mutants was significantly lower than that in the *SET1*^+^ cultures. *HIS3* mRNA levels were upregulated in the *set1-G951A* and *set1-R1013H* mutants and were not significantly different from those in the *SET1*^+^ cultures. From these results, we conclude that *set1-G951A*, an allele mainly capable of generating H3K4me1, supports the activation of transcription of the *HIS3* gene under histidine-starvation conditions. Moreover, the growth defects observed in the *set1Δ* and *set1-Y967A* cultures under histidine-starvation conditions (Fig. 2B) are likely to be due to defective induction of *HIS3*. Even though the average *HIS3:ACT1* level in the *set1-R1013H* mutant under histidine-starvation conditions was higher than that from the *set1Δ* and *set1-Y967A* cultures, the level was not statistically different from either the *SET1*^+^ or *set1Δ* and *set1-Y967A* mutants. This situation was most likely caused by variation among the five samples used to analyze the *HIS3:ACT1* level in the *set1-R1013H* mutant.

### Histone H3K4 methylation patterns indicate that H3K4me1 supports induction of the *HIS3* gene during histidine starvation

**Fig. 6:**
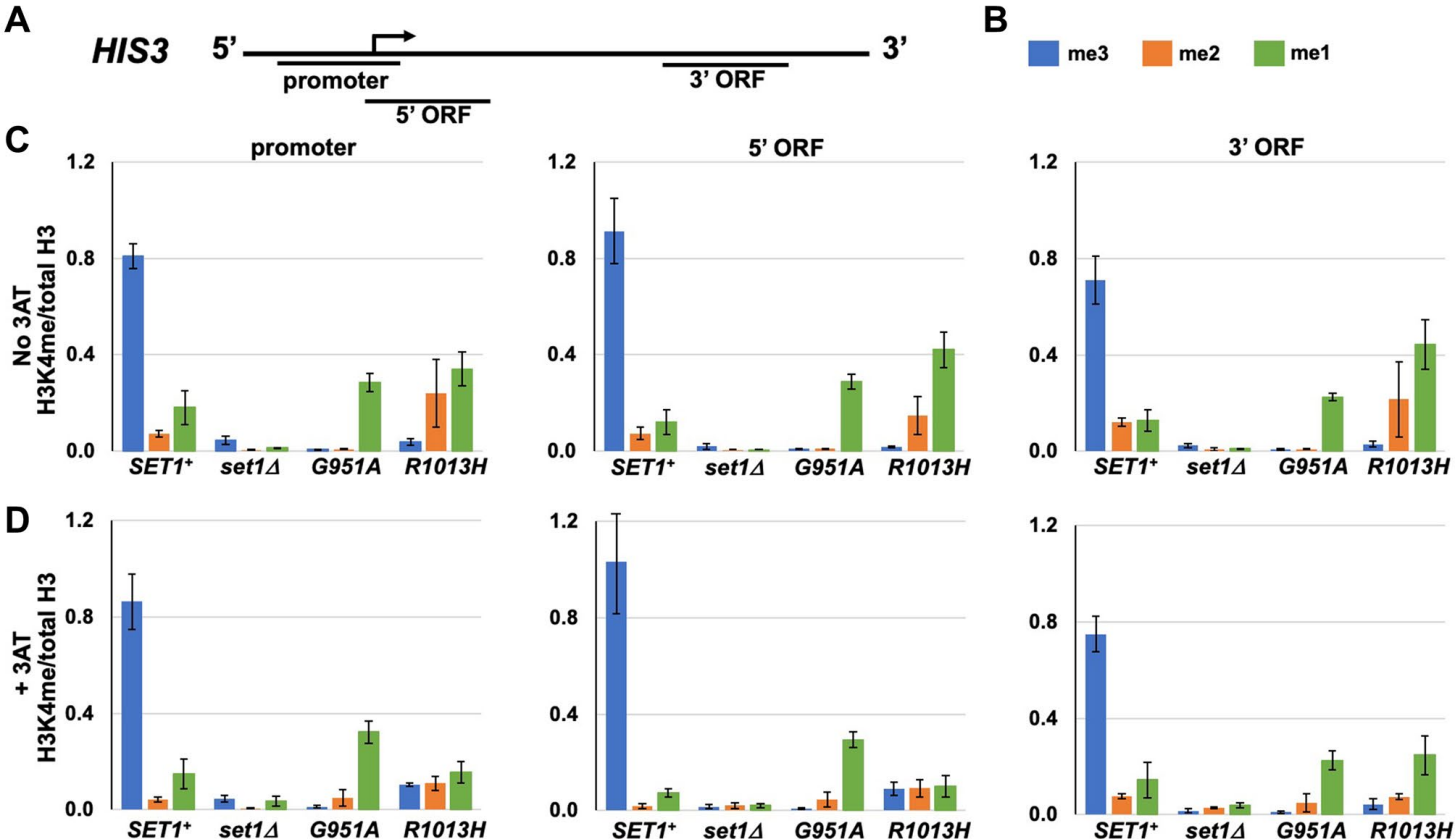
Distribution of H3K4me marks at three positions of the *HIS3* gene. **A** Schematic showing the regions of *HIS3* gene (promoter, 5′ ORF and 3′ ORF) evaluated by qPCR after ChIP for H3K4me1, H3K4me2 and H3K4me3. **B** Key to graph. **C** Association of H3K4me at the *HIS3* promoter, 5′ ORF and 3′ ORF in yeast cultures grown in SC-His. **D** Association of H3K4me at the *HIS3* promoter, 5′ ORF and 3′ ORF in yeast cultures grown in SC-HIS + 10 mM 3AT. See Supplementary Table 3 for statistical analysis; error bars, (± SEM, *n* = 4)

To evaluate the presence of H3K4 methyl marks at the *HIS3* gene during histidine starvation, ChIPs were performed to measure H3K4me1, H3K4me2 and H3K4me3 at the *HIS3* promoter and the 5′ and 3′ ends of the *HIS3* ORF (Fig. 6A, B). The distribution of H3K4 methyl marks was also determined at the promoter of the *ACT1* gene and an intergenic region on chromosome *VIII* (Supplementary Fig. 3). ChIP samples from the *set1Δ* mutant were evaluated to measure background signals. Wild-type Set1 converts lower-level H3K4 methyl marks to H3K4me3. As expected, H3K4me1/2/3 marks were detected above background at the *HIS3* promoter and the 5′ and 3′ regions of the *HIS3* ORF in yeast cultures expressing wild-type *SET1*^+^ in the absence of 3AT (Fig. 6C, see Supplementary Table 3 for statistical analysis of ChIP data). H3K4me3 and H3K4me1 were detected above background at the *HIS3* promoter and the 5′ and 3′ ORF regions in *SET1*^+^ cultures grown in the presence of 3AT (Fig. 6D). H3K4me2 was detected above background at the *HIS3* promoter, but not at the 5′ and 3′ ORF regions in *SET1*^+^ cultures grown with 3AT. In accordance with previously published data (Pokholok et al. 2005), in the *SET1*^+^ cultures, the average level of H3K4me3 was higher at the *HIS3* promoter and 5′ ORF than the 3′ ORF. The resemblance of the ChIP results with previous work will be explored in the discussion.

Consistent with the results of Western blots (Fig. 1), in chromatin from the *set1-G951A* strain, H3K4me1 was the predominant form of K4-methylated H3 at the genomic positions tested. H3K4me1 was detected above background at the *HIS3* promoter, the *HIS3* 5′ and 3′ ORF regions and the *ACT1* promoter in the *set1-G951A* mutant in the absence and presence of 3AT (Fig. 6C, D, Supplementary Fig. 3). Likewise, H3K4me1 and H3K4me2 were detected above background at the *HIS3* promoter and the 5′ and 3′ ORF regions in the *set1-R1013H* mutant in absence of 3AT (Fig. 6C). For the most part, the association of H3K4me3 at the three regions of *HIS3* gene, the *ACT1* promoter and the intergenic region in the *set1-G951A* and *set1-R1013H* mutants grown with or without 3AT were similar to background (Fig. 6C, D, Supplementary Fig. 3). An exception was seen at the *HIS3* promoter in the *set1-R1013H* cultures grown in the presence of 3AT where the H3K4me3 level was above background (Fig. 6D). The level of H3K4me2 at the *HIS3* promoter was also above background in *set1-R1013H* cultures grown in 3AT (Fig. 6D). The increase in H3K4me2 and H3K4me3 at the *HIS3* promoter in the s*et1-R1013H* mutant grown in 3AT may reflect an increase in transcription by Pol II when cells are starved for histidine. The result also indicates that the HMTase encoded by the s*et1-R1013H* allele is able to produce H3K4me3 at low levels.

It was surprising that the levels of H3K4me1, H3K4me2 and H3K4me3 associated with the *HIS3* promoter, 5′ ORF and 3′ ORF in wild-type *SET1*^+^ cultures were not significantly increased in cultures grown in presence of 3AT, conditions that we know cause an increase in steady-state *HIS3* mRNA (Fig. 5, Supplementary Table 3). Likewise, the levels of H3K4me1/2/3 associated with the *HIS3* promoter, 5′ ORF and 3′ ORF in the *set1-G951A* mutant were similar in cultures grown with and without 3AT. These results demonstrate that major changes in the levels of H3K4me3, H3K4me2 and H3K4me1 at the *HIS3* promoter, 5′ ORF and 3′ ORF are not necessary for induction of the *HIS3* gene during histidine starvation caused by treatment with 3AT. Two statistically significant differences in H3K4 methylation were detected in the *set1-R1013H* mutant when comparing ChIP data from cultures growth with 3AT to those grown without 3AT: the level of H3K4me3 was higher at the *HIS3* promoter and the level of H3K4me1 was lower at the *HIS3* 5′ ORF (Fig. 6). Gene expression and ChIP experiments focusing on genes other than *HIS3* should provide clarity regarding the function of *set1-R1013H* mutant.

The growth phenotypes observed in a *set1Δ* mutant indicate that H3K4 methylation is required for robust growth and induction of *HIS3* transcription during histidine starvation. The results show that wild-type growth and induction of the *HIS3* gene occurs in the *set1-G951A* mutant despite a lack of H3K4me3 at the *HIS3* promoter and 5′ ORF. From the data, we conclude that H3K4me1 supports transcription of the *HIS3* gene under histidine-starvation conditions and the response does not rely on H3K4me2 and H3K4me3. The implications of these findings with respect to transcription are discussed below.

## Discussion

Studies in *S. cerevisiae* have shown that H3K4me1 inhibits RSC-independent chromatin remodeling thereby preventing the induction of osmostress genes (Nadal-Ribelles et al. 2015). In higher eukaryotes, H3K4me1 is associated with transcriptional silencing (Cheng et al. 2014). In contrast to these repressive roles, the work here using partial-function variants of the HMTase Set1 shows that H3K4me1 supports activation of Pol II transcription when *S. cerevisiae* is subjected to nutrient starvation. Growth defects observed in a *set1Δ* mutant under amino-acid starvation are rescued by an H3K4me1-proficient allele, *set1-G951A*. In the *set1-G951A* mutant, H3K4me1 is the predominant H3K4 methyl mark at the *HIS3* promoter during gene induction and H3K4me3 is not detected (Figs. 1, 5, 6). Therefore, activation of the *HIS3* gene in the *set1-G951A* mutant cannot be attributed to the accumulation of H3K4me3 at the *HIS3* promoter. These findings indicate that H3K4me1 supports induction of the *HIS3* gene in the absence of higher-order H3K4 methylation. It is possible that other genes required for histidine biosynthesis are also regulated by H3K4me1. These genes will be investigated in future.

Growth defects were detected in *set1Δ* mutants grown under isoleucine–valine starvation conditions, and these were rescued by the *SET1*^+^, *set1-G951A*, *set1-R1013H* alleles, but not by *set1-Y967A*, which is defective for H3K4 methylation (Fig. 3). The data suggest that induction of at least one of the genes required for the biosynthesis of isoleucine and valine occurs in cells with H3K4me1 that lack H3K4me3. In future, *S. cerevisiae* genes that are regulated by H3K4me1 will be identified on a genome-wide scale using the *set1-G951A* mutant in RNA-seq and ChIP-seq experiments.

The three states of H3K4 methylation play different roles in gene expression (Kusch 2012; Pokholok et al. 2005). Much of the existing literature on H3K4 methylation focuses on the role of H3K4me3 in active transcription (Kusch 2012; Musselman et al. 2012; Pray-Grant et al. 2005; Schneider et al. 2005; Taverna et al. 2006), although there are several examples of genes that require H3K4me3 for repression (for example see, Carvin and Kladde 2004; Weiner et al. 2012)). As expected from previous work (Liu et al. 2005; Pokholok et al. 2005; Soares et al. 2017), ChIP analysis of the *HIS3* gene showed that the three forms of K4-methylated H3 are associated with the *HIS3* promoter and the 5′ and 3′ regions of the *HIS3* ORF in *SET1*^+^ cultures. The highest H3K4me3 signals were at the promoter and 5′ end of the *HIS3* ORF. H3K4me2 levels were lower than those of H3K4me3 and H3K4me1, and H3K4me1 levels were lower than those of H3K4me3 (Fig. 6). The H3K4 methylation profile of the *HIS3* gene in *SET1*^+^ cultures (Fig. 6) is similar to the profiles reported in previous publications (Ramakrishnan et al. 2016; Soares et al. 2017).

Histidine starvation triggered by 3AT causes an increase in *HIS3* mRNA (Hill et al. 1986). The work here shows that after one hour of induction of histidine starvation, there was a greater than 13-fold increase in *HIS3* mRNA in *SET1*^+^ cultures (Fig. 5). It is surprising that this increase in *HIS3* mRNA was not accompanied by an increase in the levels of H3K4 methyl marks at the *HIS3* promoter in *SET1*^+^ cultures (Fig. 6C, D). Based on these findings, we conclude that H3K4 methylation alone is unlikely to determine the transcriptional response to histidine starvation at the *HIS3* gene. Changes in the levels of other histone modifications, such as acetylation, in combination with H3K4 methylation, are likely to be required to recruit transcription effectors responsible for induction of the *HIS3* gene under histidine-starvation conditions. This possibility is reminiscent of the role of H3K4me1 at enhancers and promoters in eukaryotic cells.

H3K4me1 is found at enhancers and precedes acetylation of H3K27 (Calo and Wysocka 2013). These chromatin marks support enhancer–promoter interactions and transcription of enhancer RNAs (Creyghton et al. 2010; Kang et al. 2021). H3K4me1 promotes interactions between the enhancers and gene promoters by facilitating the binding of chromatin remodelers (Local et al. 2018; Yan et al. 2018). Surprisingly, catalytically defective COMPASS-like HMTases in mammals have also been shown to facilitate enhancer–promoter interactions, suggesting that is an H3K4me1-independent mechanism that supports enhancer-mediated effects on gene expression (Dorighi et al. 2017; Rickels et al. 2017). Unlike catalytically defective Set1-like HMTases at enhancers, the methylation-defective *set1-Y967A* allele did not support induction of *HIS3* (Fig. 5) and behaved like the *set1Δ* mutant (Figs. 1, 2, 3, 4, 5). Previous work suggested that the *set1-Y967A* mutant is unable to methylate Dam1, a non-histone target of the Set1, despite the presence of wild-type levels of Set1-Y967A protein in whole cell extracts (Williamson et al. 2013). The results obtained with the *set1-Y967A* mutant support the conclusion that Set1 HMTase activity is required for upregulation of *HIS3* gene expression during histidine starvation.

The association of H3K4me1 with enhancers in higher eukaryotes is well established. Recent work revealed that H3K4me1 at promoters in human and murine germ cells can provide information about the transcription potential of a gene (Bae and Lesch 2020). The authors concluded that H3K4me1 found together with H3K4me3 and H3K27me3 denotes a transcriptionally poised promoter and they speculated that the presence of H3K4me1 at a poised promoter may reduce the action of DNA methyltransferases, essentially providing a mechanism to maintain the promoter in a neutral state that can be activated. Other studies have shown that H3K4me1 is correlated with regions of intermediate DNA methylation (Sharifi-Zarchi et al. 2017). A clear picture of the functions of H3K4me1 at promoters is still emerging.

The initiation of Pol II transcription requires the assembly of transcription factors at gene promoters. At *HIS3* and other genes, Gcn4 recruits the SAGA complex (Spt-Ada-Gcn5 acetyltransferase) to gene promoters (Kuo et al. 2000). Gcn4 also interacts with SWI/SNF, the SRB/Mediator complex, RNA polymerase II, TFIID, and NuA4 (Ginsburg et al. 2009; Natarajan et al. 1999; Swanson et al. 2003). Identification of proteins in *S. cerevisiae* that read H3K4me1 will help elucidate H3K4me1-dependent mechanisms of transcription activation. Previous work in human cells showed that the chromodomain of the acetyltransferase, Tip60, recognizes H3K4me1 at certain enhancer elements (Jeong et al. 2011). In future, we will identify transcriptional effectors that read H3K4me1 and determine if these contribute to the activation of transcription at *HIS3* and other genes.

H3K4 methyltransferases are conserved across species from yeast to humans and have important roles in regulation of gene expression (reviewed in Shilatifard (2012)). The involvement of H3K4me3 in gene activation (Pray-Grant et al. 2005) and repression (Shi et al. 2006) illustrates the complexity of the regulation mediated by Set1 and Set1-like methyltransferase. The identification and characterization of H3K4me1-mediated mechanisms will contribute to our understanding of transcriptional regulation and gene expression.

## Supplementary Information

Below is the link to the electronic supplementary material.

Supplementary file1 (DOCX 5003 KB)

## Data Availability

The authors will make all data and unique research materials and data freely available to other investigators.

## Abbreviations

H3K4: Histone H3 lysine 4
H3K4me1: K4-monomethylated histone H3
H3K4me2: K4-dimethylated histone H3
H3K4me3: K4-trimethylated histone H3
HAT: Histone acetyltransferase
ORFs: Open reading frames
3AT: 3-Amino-1,2,4-triazole
