# Supplementary material for: Histone 3 lysine 4 monomethylation supports activation of transcription in S. cerevisiae during nutrient stress
Source: Curr Genet. 2022 Jan 18;68(2):181–94. doi: 10.1007/s00294-022-01226-2 (PMC8976815; doi:10.1007/s00294-022-01226-2)
Supplement: Supplementary file 1 — Supplementary file1 (DOCX 5003 KB) [file 294_2022_1226_MOESM1_ESM.docx]

Supplementary Materials for

**Histone 3 Lysine 4 monomethylation supports gene activation in *S. cerevisiae* under nutrient stress**

Neha Deshpande, Rachel Jordan, Michelle Henderson Pozzi, and Mary Bryk

Supplementary Table 1 Yeast Strains

Supplementary Table 2 Oligonucleotides

Supplementary Table 3 Statistical Analysis of Chromatin IPs

Supplementary Fig 1 Time-course of induction of *HIS3* transcript with 10 mM 3AT

Supplementary Fig 2 Methylation of histone H3K4 by Set1 is required for robust growth during isoleucine valine starvation

Supplementary Fig 3 Distribution of H3K4me marks at the promoter of the *ACT1* gene and an intergenic region

**Supplementary Table 1 Yeast Strains**

| **Strain** | **Genotype** | **Reference** |
| --- | --- | --- |
| MBY1590 | *MATa ura3-52 trp1Δ63 set1Δ::KANMX4 lys2-128δ* | This study |
| MBY2992 | *MATa ura3-52::pRS406 trp1Δ63 set1Δ::KANMX4 lys2-128δ* | This study |
| MBY2994 | *MATa ura3-52::pRS406-SET1 trp1Δ63 set1Δ::KANMX4 lys2-128δ* | This study |
| MBY2996 | *MATa ura3-52::pRS406-set1-R1013H trp1Δ63 set1Δ::KANMX4 lys2-128δ* | This study |
| MBY2998 | *MATa ura3-52::pRS406-set1-Y967A trp1Δ63 set1Δ::KANMX4 lys2-128δ* | This study |
| MBY3029 | *MATa (hht1-hhf1)Δ::LEU2 lys2-128δ trp1Δ63 ura3-52 set1Δ::KANMX4 (hht2-hhf2)Δ::KANMX4 pHHT2-HHF2-TRP1 CEN (leu2Δ1 or LEU2)* | This study |
| MBY3030 | *MATa (hht1-hhf1)Δ::LEU2 lys2-128δ trp1Δ63 ura3-52 set1Δ::KANMX4 (hht2-hhf2)Δ::KANMX4 phht2-K4R-HHF2-TRP1 CEN (leu2Δ1 or LEU2)* | This study |
| MBY3031 | *MATa (hht1-hhf1)Δ::LEU2 lys2-128δ trp1Δ63 ura3-52::pRS406-SET1 set1Δ::KANMX4 (hht2-hhf2)Δ::KANMX4 pHHT2-HHF2-TRP1 CEN (leu2Δ1 or LEU2)* | This study |
| MBY3032 | *MATa (hht1-hhf1)Δ::LEU2 lys2-128δ trp1Δ63 ura3-52::pRS406 set1Δ::KANMX4 (hht2-hhf2)Δ::KANMX4 pHHT2-HHF2-TRP1 CEN (leu2Δ1 or LEU2)* | This study |
| MBY3033 | *MATa (hht1-hhf1)Δ::LEU2 lys2-128δ trp1Δ63 ura3-52::pRS406-set1-Y967A set1Δ::KANMX4 (hht2-hhf2)Δ::KANMX4 pHHT2-HHF2-TRP1 CEN (leu2Δ1 or LEU2)* | This study |
| MBY3035 | *MATa (hht1-hhf1)Δ::LEU2 lys2-128δ trp1Δ63 ura3-52::pRS406-set1-R1013H set1Δ::KANMX4 (hht2-hhf2)Δ::KANMX4 pHHT2-HHF2-TRP1 CEN (leu2Δ1 or LEU2)* | This study |
| MBY3037 | *MATa (hht1-hhf1)Δ::LEU2 lys2-128δ trp1Δ63 ura3-52 ::pRS406-SET1 set1Δ::KANMX4 (hht2-hhf2)Δ::KANMX4 phht2-K4R-HHF2-TRP1 CEN (leu2Δ1 or LEU2)* | This study |
| MBY3038 | *MATa (hht1-hhf1)Δ::LEU2 lys2-128δ trp1Δ63 ura3-52 ::pRS406 set1Δ::KANMX4 (hht2-hhf2)Δ::KANMX4 phht2-K4R-HHF2-TRP1 CEN (leu2Δ1 or LEU2)* | This study |
| MBY3039 | *MATa (hht1-hhf1)Δ::LEU2 lys2-128δ trp1Δ63 ura3-52 ::pRS406-set1-Y967A set1Δ::KANMX4 (hht2-hhf2)Δ::KANMX4 phht2-K4R-HHF2-TRP1 CEN (leu2Δ1 or LEU2)* | This study |
| MBY3041 | *MATa (hht1-hhf1)Δ::LEU2 lys2-128δ trp1Δ63 ura3-52 ::pRS406-set1-R1013H set1Δ::KANMX4 (hht2-hhf2)Δ::KANMX4 phht2-K4R-HHF2-TRP1 CEN (leu2Δ1 or LEU2)* | This study |
| MBY3078 | *MAT alpha leu2Δ1 lys2-128δ ura3-52 trp1Δ63 set1Δ::TRP1* | This study |
| MBY3148 | *MAT alpha leu2Δ1 lys2-128δ ura3-52::pRS406-SET1 trp1Δ63 set1Δ::TRP1* | This study |
| MBY3149 | *MAT alpha leu2Δ1 lys2-128δ ura3-52::pRS406 trp1Δ63 set1Δ::TRP1* | This study |
| MBY3152 | *MAT alpha leu2Δ1 lys2-128δ ura3-52::pRS406-set1-Y967A trp1Δ63 set1Δ::TRP1* | This study |
| MBY3154 | *MAT alpha leu2Δ1 lys2-128δ ura3-52::pRS406-set1-G951A trp1Δ63 set1Δ::TRP1* | This study |
| MBY3181 | *MAT alpha leu2Δ1 lys2-128δ ura3-52::set1-R1013H trp1Δ63 set1Δ::TRP1* | This study |
| MBY3242 | *MATa (hht1-hhf1)Δ::LEU2 lys2-128δ trp1Δ63 ura3-52::pRS406-set1-G951A set1Δ::KANMX4 (hht2-hhf2)Δ::KANMX4 pHHT2-HHF2-TRP1 CEN (leu2Δ1 or LEU2)* | This study |
| MBY3243 | *MATa (hht1-hhf1)Δ::LEU2 lys2-128δ trp1Δ63 ura3-52 ::pRS406-set1-G951A set1Δ::KANMX4 (hht2-hhf2)Δ::KANMX4 phht2-K4R-HHF2-TRP1 CEN (leu2Δ1 or LEU2)* | This study |

**Supplementary Table 2. Oligonucleotides**

| **Oligo name** (Collection number) | **Sequence** |
| --- | --- |
| HIS3 promoter FP (OM18) | TTG-GCC-TCC-TCT-AGT-ACA-CTC |
| HIS3 promoter RP (OM1212) | ACT-AGG-GCT-TTC-TGC-TCT-GTC |
| HIS3 5' ORF FP (OM1262) | GCA-GGC-AAG-ATA-AAC-GAA-GGC |
| HIS3 5' ORF RP (OM19) | GGT-CCA-GAA-ACC-CTA-TAC-CTG |
| HIS3 3' ORF FP (OM1101) | TGA-TCA-TCA-CCG-TAG-TGA-GAG |
| HIS3 3' ORF RP (OM1102) | GCA-TTA-CCT-TGT-CAT-CTT-CAG |
| ACT1 promoter FP (OM1260) | CTT-CCC-CTT-TCT-ACT-CAA-ACC |
| ACT1 promoter RP (OM1261) | CGC-TAG-AAC-ATA-CCA-GAA-TCC |
| IGR FP (OM482) | CAG-TCA-ATC-AGC-GTA-GTG-AC |
| IGR RP (OM483) | CAT-TCG-GGC-AGA-ACT-GTA-AAC |

**Supplementary Table 3. Statistical Analysis of Chromatin IPs**

**Supplementary Fig 1**
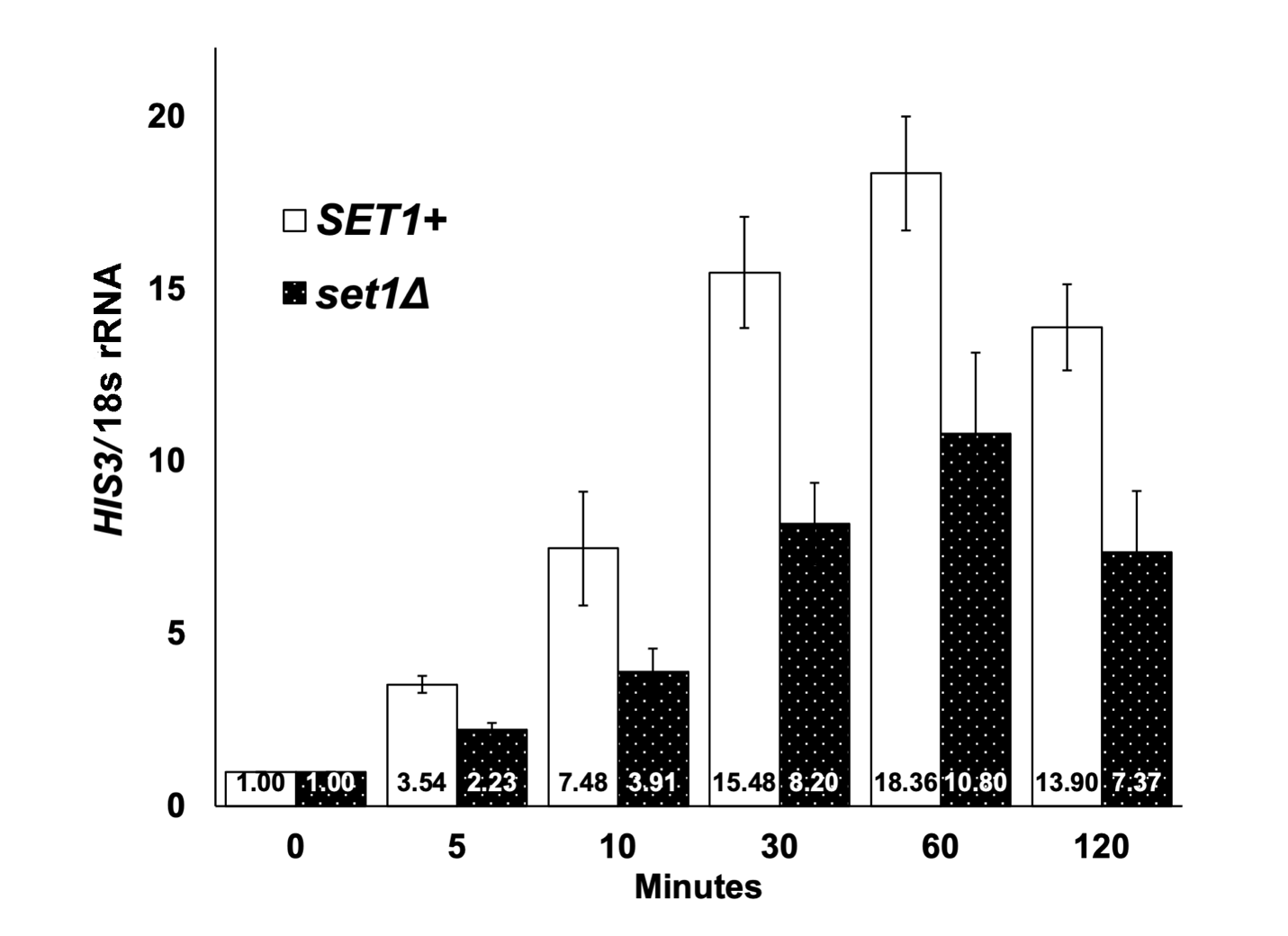


**Supplementary Fig 1 Time-course of induction of *HIS3* transcript with 10 mM 3AT** Cultures of *SET1^+^* (MBY2994) and *set1Δ* (MBY2992) yeast strains were grown to log phase in SC-His followed by addition of 10 mM 3AT to induce *HIS3* gene expression. *HIS3* mRNA levels over time after 3AT induction were analyzed by Northern blot. Average *HIS3* mRNA levels were normalized to 18S ribosomal RNA. Each bar represents the ratio of (*HIS3/rRNA)* and the error bars (+/-SEM, n=3). The *HIS3* transcript level increased steadily, reaching a peak at 60 min after addition of 10 mM 3AT, and then decreased over the remainder of the time course. This pattern was also observed in the *set1Δ* culture, but the amount of *HIS3* mRNA was lower than in the corresponding *SET1^+^* samples.

**Supplementary Fig 2**


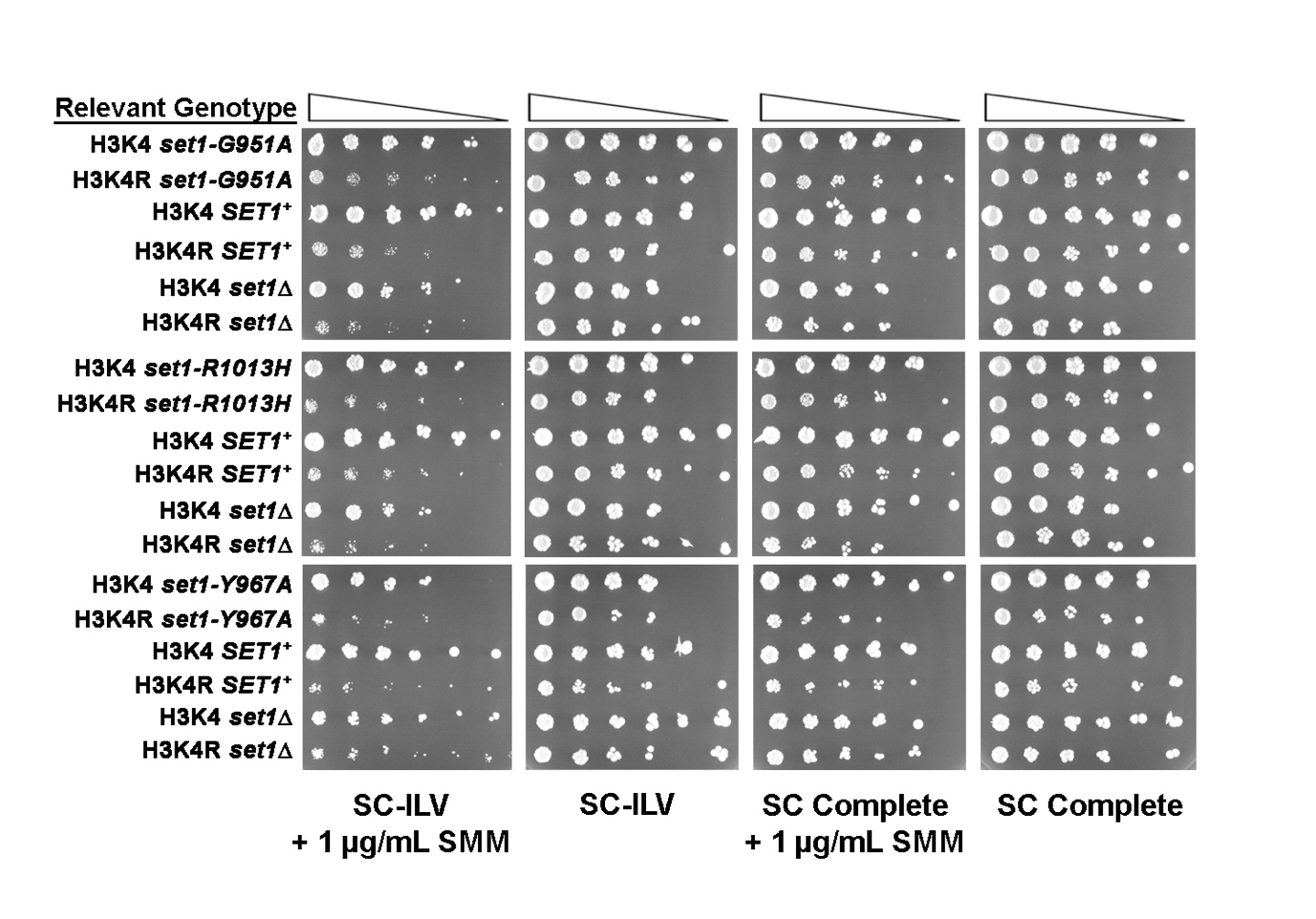


**Supplementary Fig 2 Methylation of histone H3K4 by Set1 is required for robust growth during isoleucine valine starvation** Five-fold serial dilutions of yeast strains expressing wild type *SET1* or mutant *set1* alleles were spotted onto SC-Ile Val or SC Complete solid agar with 1 µg/mL SMM or DMSO. The relevant genotypes listed on the left indicate yeast strains expressing wild-type or mutant alleles of *SET1* and either wild-type histones H3 and H4 (H3K4) or mutant H3 K4R and wild-type H4, (H3K4R). Plates were incubated at 30^o^C for 4-5 days prior to imaging. Other labels, as in Figure 2.

**Supplementary Fig 3**


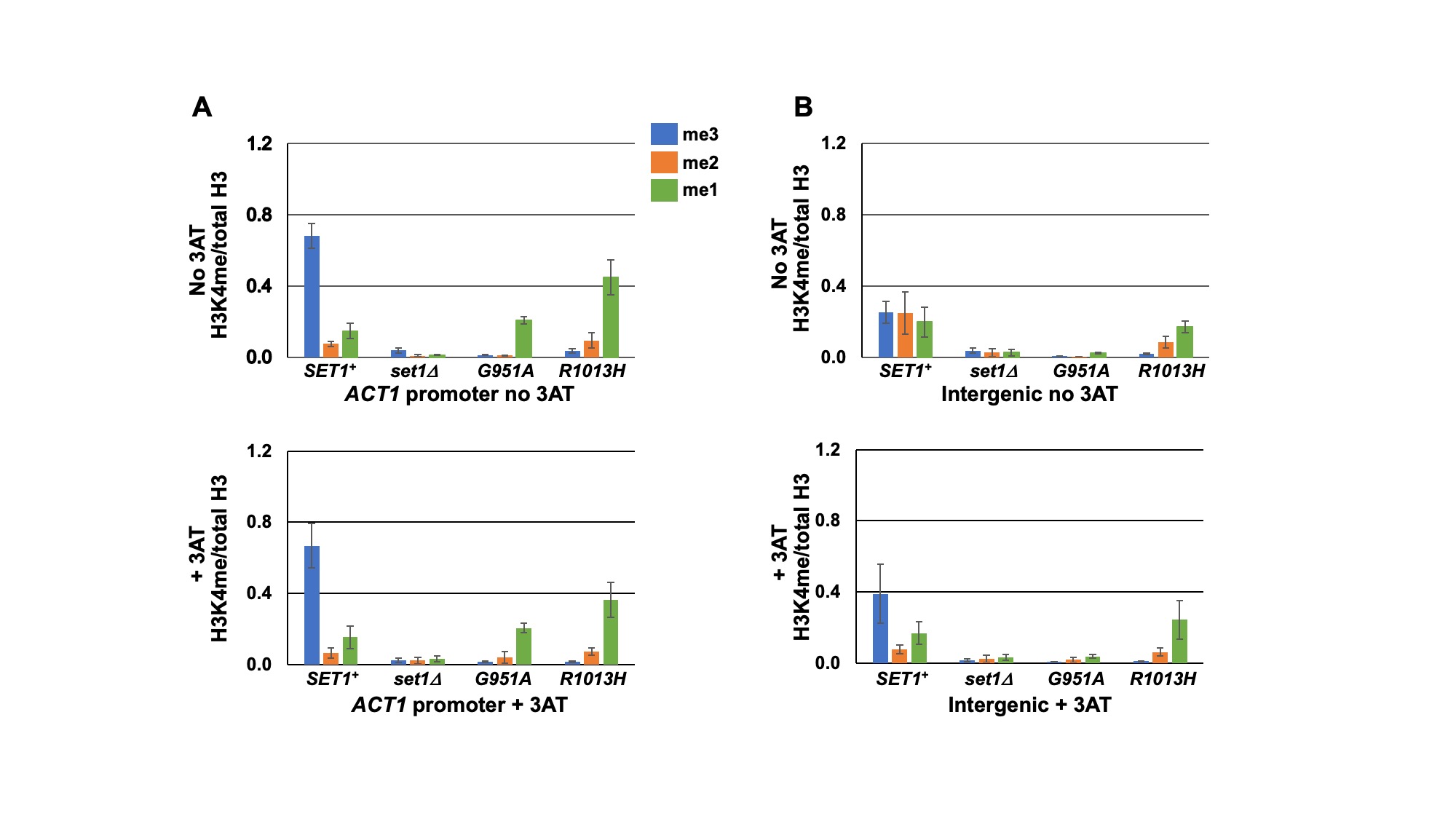


**Supplementary Fig 3** **Distribution of H3K4me marks at the promoter of the *ACT1* gene and an intergenic region**. A. H3K4me1, H3K4me2 and H3K4me3 measured at the *ACT1* promoter in yeast cultures grown in SC-HIS (upper) and SC-HIS+10 mM 3AT (lower). B. Levels of H3K4me1, H3K4me2 and H3K4me3 measured at an intergenic region on *S. cerevisiae* chromosome VIII from cultures grown in SC-HIS (upper) and SC-HIS+10 mM 3AT (lower). Percent IPs were normalized to total H3. Error bars (+/- SEM, n=4).
